# Intravascular Large B-Cell Lymphoma of the Gallbladder

**DOI:** 10.4274/tjh.2017.0276

**Published:** 2018-05-25

**Authors:** Bülent Çetin, Nalan Akyürek, Yavuz Metin, Feryal Karaca, İrem Bilgetekin, Ahmet Özet

**Affiliations:** 1 Recep Tayyip Erdoğan University Faculty of Medicine, Department of Internal Medicine, Division of Medical Oncology, Rize, Turkey; 2 Gazi University Faculty of Medicine, Department of Pathology, Ankara, Turkey; 3 Recep Tayyip Erdoğan University Faculty of Medicine, Department of Radiology, Rize, Turkey; 4 Adana Numune Training and Research Hospital, Clinic of Radiation Oncology, Adana, Turkey; 5 Dr. Abdurrahman Yurtaslan Ankara Oncology Training and Research Hospital, Clinic of Internal Medicine, Division of Medical Oncology, Ankara, Turkey; 6 Gazi University Faculty of Medicine, Department of Internal Medicine, Division of Medical Oncology, Ankara, Turkey

**Keywords:** Intravascular large B-cell lymphoma, Gallbladder, Gastrointestinal stromal tumor

**To the Editor,**

Intravascular large B-cell lymphoma (IVLBCL) is a rare type of extranodal B-cell lymphoma characterized by the growth of lymphoma cells within the lumina of small vessels. Two major patterns of clinical presentation have been recognized: the first is in European countries, with brain and skin involvement, and the second in Asian countries, where patients typically present with multiorgan failure, hepatosplenomegaly, pancytopenia, and hemophagocytic syndrome [1,2,3,4,5]. Primary IVLBCL of the gallbladder is exceedingly rare.

A 60-year-old male patient was admitted to the hospital with fever, abdominal pain, and weight loss. Physical examination showed an epigastric mass of approximately 4 cm in diameter and the absence of hepatosplenomegaly and lymphadenopathy. Laboratory tests revealed anemia (hemoglobin: 10 g/dL), with normal leukocytes and platelets. Peripheral smear showed normocytic-normochromic anemia without any abnormal cells. Increases in liver function tests were positive laboratory findings (aspartate aminotransferase: 240 U/L, alanine aminotransferase: 240 U/L, alkaline phosphatase: 740 U/L, gamma-glutamyl transferase: 80 U/L, total bilirubin/direct bilirubin: 2.06/1.2 mg/dL). Contrast-enhanced abdominal computerized tomography (CT) for further evaluation revealed a greater curvature-based mass of 8x5x5.5 cm in size, at the level of the distal gastric corpus, significantly narrowing the gastric lumen (Figures 1A and 1B). CT also showed hypodense areas in liver segments 5 and 8 and gallbladder stones, the largest being 1.5 cm in diameter. Dynamic liver magnetic resonance imaging (MRI) was performed to characterize the liver lesions. MRI revealed calculous cholecystitis, choledocholithiasis, and a mass lesion of 6.5x3 cm in size, thought to be based on the greater curvature at the corpus of the stomach. With no signs of distant metastasis, the patient primarily underwent both cholecystectomy and partial gastrectomy. Surgical biopsy of liver lesions revealed nonspecific inflammatory changes and no evidence of a tumor, while histologic examination confirmed a gastrointestinal stromal tumor (GIST) of the stomach. Histological analysis of the cholecystectomy material showed cells with irregular nuclear contours and open chromatin confined to small vessels, characteristic of the IVLBCL phenotype. These cells were strongly positive for CD20 stain (Figures 1C and 1D). Since intravascular infiltrations are easily missed on hematoxylin and eosin-stained sections, bone marrow and liver biopsy slides were also stained by CD20 and no evidence of intravascular lymphoma was found. A whole-body integrated positron electron tomography-CT scan for tumor staging showed diffusely increased uptake of 18F-fludeoxyglucose in the liver (SUV_max_: 7.0) and multiple lymph node lesions including the submandibular, preauricular, cervical, and jugular lymph nodes (SUV_max_: 8.3). He was treated with six cycles of an R-CHOP regimen. He did not show any evidence of recurrence (normal gastroscopy and CT scan) at 36 months of follow-up.

IVLBCL usually occurs in adults in the sixth and seventh decades. The tumor is often clinically unsuspected and can be easily overlooked on biopsy. The diagnosis is most commonly made at autopsy. The lymphoma cells are generally large with round nuclei and prominent nucleoli. The malignant cells uniformly express pan-B-cell antigens (CD20, CD79a) and variably express other antigens such as CD5 (38%) and CD10 (13%) [2]. There are no pathognomonic laboratory or radiologic abnormalities associated with IVLBCL. Abdominal CT and MRI findings of our patient with IVLBCL were nonspecific. What is the pathogenic mechanism for simultaneous presentation of gallbladder intravascular B-cell lymphoma with GIST? A unifying hypothesis supports a single underlying genetic instability that could have led to both diseases. The finding of two different neoplasms in our patient seems to be coincidental rather than related to the same pathogenic triggering. Central nervous system symptoms, skin manifestations, bone marrow involvement, and hemophagocytic syndrome are the most common clinical and laboratory abnormalities, but these were not seen in our case. Our patient presented with nonspecific symptoms and laboratory abnormalities. The ability of IVLBCL to involve any organ system further makes it very difficult to suspect this condition in a patient with a rare presentation such as ours.

## Figures and Tables

**Figure 1:**
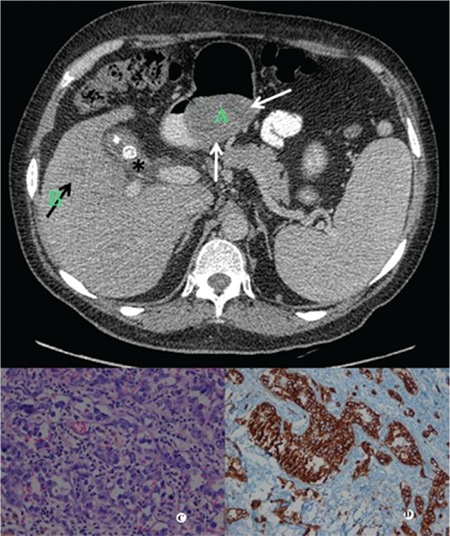
A, B) Axial computerized tomography image showing a greater curvature-based intraluminal gastric mass (white arrows), stones in the gallbladder (asterisk), and a vague hypodense area, which was proven to be caused by cholangitis, in segment 5 of the liver (black arrow). C) Intravascular B-cell lymphoma. The numerous dilated blood vessels were filled with large, atypical, centroblast-like lymphoid cells (hematoxylin and eosin, 400^x^). D) CD20-positive atypical lymphoid cells (400^x^).
